# A Scoping Review of the Impact of Theatre-Based Techniques in Children and Adults with Autism Spectrum Disorders

**DOI:** 10.3390/bs16060890

**Published:** 2026-06-01

**Authors:** Angelos Papadopoulos, Vasiliki Zarokanellou, Nikolaos Psylakis, Dionysios Tafiadis, Angeliki Tsapara, Nikolaos Trimmis, Panagiotis Plotas

**Affiliations:** 1 Department of Speech and Language Therapy, School of Health Sciences, University of Ioannina, 45500 Ioannina, Greece; vzarokanellou@uoi.gr (V.Z.); psilakis1@gmail.com (N.P.); tafiadis@uoi.gr (D.T.); 2 Department of Speech and Language Therapy, School of Health Rehabilitation Sciences, University of Patras, 26504 Patras, Greece; up1090680@upatras.gr (A.T.); nicktrimmis@upatras.gr (N.T.)

**Keywords:** theatre-based techniques, dramatherapy, scoping review, social skills, children, adults, Autism, ASD

## Abstract

(1) Background: The present scoping review sought to map the characteristics of theatre-based interventions and their reported effects on social interactions, social communication, anxiety, and broader psychosocial functioning of individuals with Autism Spectrum Disorder (ASD). (2) Methods: A search was conducted in the Scopus, ERIC, and PubMed databases, and manually with specific terms under PRISMA guidelines and the PCC framework. Eleven studies met the inclusion criteria. (3) Results: Most of the studies were conducted in the United States over the past 15 years. The interventions were categorized according to the method used in each study. All the studies included children and adolescents as samples, with only one study including adults. The SENSE Theatre Program was the most frequently applied method. There was some heterogeneity in the duration and number of sessions applied. Improvements were observed in social cognition, behavior, interaction, social and psychological functioning, language-related areas, and the maintenance and generalization of acquired skills, with a reduction in anxiety levels. (4) Conclusions: Although the studies were limited, theatre-based interventions and dramatherapy appear to be a promising therapeutic approach that remains largely unexplored. Further research is crucial to enriching the existing literature, filling current gaps, and establishing a protocol that integrates the aforementioned interventions into clinical practices for both children and adults with ASD.

## 1. Introduction

According to the fifth edition of the Diagnostic and Statistical Manual of Mental Disorders (DSM-5), autism is defined as a neurodevelopmental disorder that impacts multiple aspects of a child’s life (Corbett et al., 2014b; Stratou et al., 2023; Wang & Lee, 2020). It is a neurodevelopmental condition characterized by difficulties in social communication and interaction, challenges in forming relationships, restricted and repetitive patterns of behavior, and heightened levels of anxiety, particularly in social contexts (American Psychiatric Association, 2013). These difficulties often affect autonomy and motivation for socialization, making everyday functioning more demanding (Corbett et al., 2014b; Stratou et al., 2023; Wang & Lee, 2020). DSM-5 classifies ASD based on the severity of presented symptoms into three different levels: Level 1—“requiring support”, Level 2—“requiring substantial support” (significant difficulties), and Level 3—“requiring special substantial support” (severe difficulties in socialization and flexibility) (Lohr & Tanguay, 2013; Lord & Bishop, 2015; Volkmar & McPartland, 2014).

Although the exact causes of ASD remain unknown, they are considered to result from neurobiological abnormalities (Bhat et al., 2014), which are associated with genetic, environmental, and hereditary factors (Johnson & Myers, 2007). In a 2020 study, the estimated prevalence of Autism Spectrum Disorder is approximately 1% of the global population, with males being diagnosed four times more frequently than females (Maenner et al., 2020). Autism manifests differently in girls compared to boys (Duvekot et al., 2017), with many girls adopting “masking” behaviors-actively concealing their symptoms to appear “normal”, which often leads to delayed or no diagnosis at all (Bargiela et al., 2016; Loomes et al., 2017). Epidemiological data from the Autism and Developmental Disabilities Monitoring (ADDM) Network report an ongoing rise in the prevalence of ASD in eight-year-old children, indicating that 1:36 children have a diagnosis of ASD, leading to an increasing number of children facing social communication difficulties and elevated levels of anxiety.

The difficulties described above highlight the importance of interventions designed to strengthen social competence and, therefore, enhance social skills (Scattone, 2007). These skills comprise a set of social behaviors that can be taught and practiced, promoting successful social integration (Corbett et al., 2014a; Gonzalez-Lopez & Kamps, 1997; Scattone, 2007). Within this context, creative art therapies have emerged as particularly effective in recent years.

Creative Arts Therapies is an umbrella term covering several specialized disciplines, including theatre-based intervention and Dramatherapy, Psychodrama, Dance/Movement Therapy, Music Therapy, Art Therapy, a combination of these, and others (Shafir et al., 2020). Theatre-based interventions constitute a separate area of creative art therapies as they involve a dramatherapist who applies a variety of teaching strategies and theatre-based techniques to engage participants in active learning of social and communication skills (O’Sullivan, 2015). Theatre as an intervention relies on existing scripts or scripts the group creates, rehearses, and then performs in front of an audience, whereas drama in education focuses more on the process and improvisation than on performance (O’Sullivan, 2015). Creative drama, on the other hand, involves dramatic experiences designed for participants’ benefit, providing them with opportunities to engage in role-play, analyze roles, and work cooperatively on creative tasks that require emotional control (Freeman et al., 2003).

The British Association of Dramatherapists defines Dramatherapy as “a form of psychotherapy that combines the therapist’s knowledge of theater techniques with therapeutic practices to promote psychological well-being”. Moreover, the North American Dramatherapy Association (NADTA) defines dramatherapy as the intentional use of drama and/or theater processes to achieve therapeutic goals. As an active, embodied, and experiential approach, it provides a safe space for participants to tell stories, set goals, solve problems, express feelings, and achieve catharsis. Through drama, the depth and breadth of inner experience can be actively explored, and interpersonal relationship skills can be enhanced.

Theatre-based techniques include improvisation, script work, role-playing, storytelling and story writing, imitation, puppetry and mask work, as well as other theatrical games (Langley, 2006). All the above theatre-based techniques and interventions are implemented in groups, fostering self-definition in relation to others and thus targeting social communication and social competence. Moreover, drama games, improvisation, and performing are fun and playful; therefore, they motivate children to focus on and participate in them. Participants are encouraged to explore their physical dimension and become aware of the range of their emotions within a safe therapeutic framework, thereby developing self-awareness and a deeper understanding of others (Κουρουκλή-Ρόμπερτσον & Ρόμπερτσον, 2018). Additionally, another advantage of dramatherapy over other interventions is that it is embodied and concrete, rather than abstract and symbolic. Kids can feel the information being taught in their bodies rather than just thinking about it—and often they need new information presented in a way that they can prove to themselves that it works.

According to the British Association of Drama therapists (Iordanou, 2019), Dramatherapy and Theatre-based techniques have been used across a wide range of populations, including individuals with addiction (Iordanou, 2019; Morris, 2014), mental health conditions (Morris, 2014; Orkibi et al., 2023), anxiety (Andonov & Wolfe, 2020), dementia (Jaaniste, 2018; Li-Wei et al., 2022, p. 20), depression (Bakhtiari et al., 2020), Parkinson’s disease (Cochrane, 2022; Keisari & Palgi, 2017), personality disorders, (De Gruijter et al., 2024; Doomen, 2018), intellectual and developmental disabilities (Foloştină et al., 2015; Geiger et al., 2020; Wu et al., 2020) as well as with psychotic disorders (Melvin et al., 2024; Tang et al., 2020). Finally, current literature indicates that such interventions have also been used for individuals with ASD (Bololia et al., 2022; Corbett et al., 2014b; Martí-Vilar et al., 2023; Peter, 2003). Dramatherapy has been shown to provide models of social skills and appropriate behavioral responses. Through observational learning, these models help individuals acquire new behaviors and refine existing ones, leading to improved social functioning and reduced disruptive behaviors (Rahimi Pordanjani, 2021). Additionally, it can serve as a vehicle for various therapeutic approaches, tailored to the unique needs of each individual (Galligan, 2009).

Two distinct theatre-based interventions (SENSE Theater Program and Hunter Heartbeat Method) have been highlighted in the literature. The SENSE Theater program is an intervention grounded in theatrical techniques, incorporating key strategic principles from learning theory and the active involvement of specially trained peers. The program aims to enhance social competence skills and provide opportunities to practice them through structured theatrical games and peer interactions, thereby reducing social anxiety (Corbett et al., 2014b).

The Hunter Heartbeat Method utilizes theatrical activities inspired by William Shakespeare’s play “The Tempest”. Through rhyme and interactive games, the program aims to teach relevant skills for social functioning in individuals with ASD, including proper communication patterns, eye contact, social improvisation, humor, and turn-taking, and to improve expressive abilities (Mehling et al., 2017). It also uses A Midsummer Night’s Dream. The second is a comedy and provides lots of opportunity for the exaggeration of emotion in the voice and body (Hunter, 2025).

Moreover, SENSE is a more inclusive program, with participants with ASD working with typically developing peers and adults throughout the projects, whereas the Hunter Heartbeat Method is usually conducted with a group leader and adult assistants rather than peers.

To our knowledge, until today, no prior study has systematically mapped and synthesized the existing literature about the outcomes of theatre-based interventions on individuals with ASD. Previous systematic reviews and meta-analyses (Li-Wei et al., 2022; Martí-Vilar et al., 2023; Zhang et al., 2024) have investigated specifically the effects of theatre-based interventions on social skills of individuals with ASD or have explored the impact of art-based interventions, including music therapy, TOMATIS intervention, sand play therapy, painting-based art therapy, etc., on children and adolescents with ASD. The present scoping review sought to map the limited body of literature on this field and identify the characteristics of Theatre-Based Techniques and their preliminary outcomes across referred areas (e.g., changes in social interactions, social communication, anxiety, and broader psychosocial functioning) in individuals with ASD. Additionally, a secondary aim was to contribute to and enrich the existing literature and to support the future design of related studies.

## 2. Materials and Methods

### 2.1. Search Strategy

In March 2025, the first searches of the Eric, Scopus, and PubMed databases were conducted, with an update in March 2026. Moreover, a manual search of the studies’ reference lists was conducted. In the Eric database, the filter “peer-reviewed only” was used. Terms related to autism spectrum disorders (ASD, autism, asperger, rett, pervasive, disintegrative) and terms related to theatre-based interventions (dramatherapy, theatre techniques) were used, combined with the logical operators “AND” and “OR”. Regarding the terms associated with Autism Spectrum Disorder, the above terms were used to encompass every possible case study conducted in the past. In Table 1 below, you can see the search strategy in detail.

### 2.2. Inclusion and Exclusion Criteria

The eligibility criteria are based on the PCC (Population, Concept, Context) recommendation by the JBI guidelines as presented in Table 2 below (Peters et al., 2020). Specifically, for the selection of articles, the following inclusion criteria were set: (a) Randomized or non-randomized controlled trials, pilot, exploratory and experimental studies, (b) population of any age, (c) population diagnosed with Autism Spectrum Disorder: Asperger’s syndrome, Rett syndrome, pervasive developmental disorder, autism disorder, (d) studies using dramatherapy or therapies through theatre techniques, and (e) studies written in English. The dataset for the present study is extended, without time limitations, to include all available literature.

Exclusion criteria: (a) review studies, (b) all studies that were not published in English, (c) studies involving a population other than Autism Spectrum Disorder, (d) studies dealing with creative arts therapies outside of Dramatherapy/theatre-based approaches, (e) studies with their full text not available. No search filters were used during the search. The criteria were applied to the final search.

### 2.3. Study Selection

**Figure 1:**
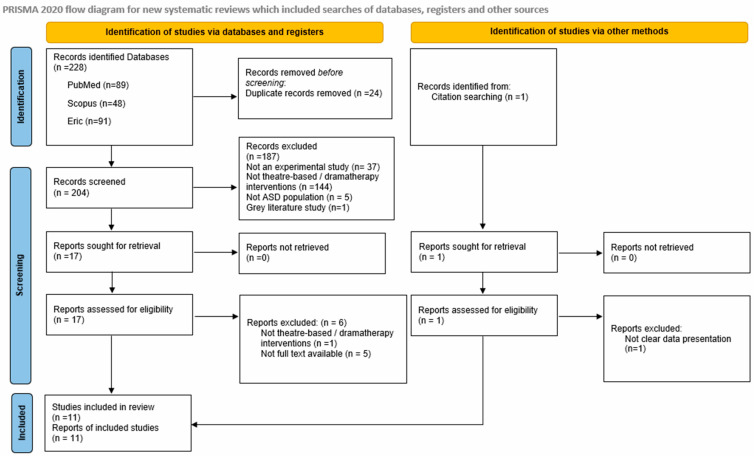
Flow chart of the process of the entire search in the databases and other sources.

This review is based on the PRISMA recommendations for systematic and scoping reviews. A flow diagram was provided to illustrate the study selection process (Figure 1). The screening and review of articles identified during the database search process were performed using Mendeley Reference Manager version 2.90.0. The database search results from Eric, Scopus, and PubMed were imported into Mendeley, and duplicates were subsequently removed. The remaining articles were screened in three stages: title, abstract, and full text. Specifically, the results from the search in the three databases totaled 227, and 1 result from other sources. Specifically, one study was identified during the hand search and assessed for eligibility using the same criteria as in the first-stage screening. After removing duplicates, the 228 remaining articles were screened based on their titles. After removing duplicates, 204 articles were screened based on titles. These articles were screened further based on their abstracts. The screening phase was performed by two independent researchers (the first and third authors of the study), and all disagreements were resolved through discussion. Finally, the full texts of 18 articles were screened for eligibility according to the study’s inclusion criteria. Of these, only 11 met the selection criteria and were included in the current scoping review. The final number of the studies included was eleven (11).

### 2.4. Data Extraction and Synthesis

Table A1 (Appendix A) was then created from the extracted data for each study included in this review. Specifically, the findings were grouped, presented, and discussed according to the key domains that emerged from the included studies. These are:

1. Studies;
2. Design;
3. Sample and Country;
4. Participants (diagnosis/classification, Age);
5. Intervention/Control Group;
6. Tools and Methods of Assessment;
7. Study Results.

### 2.5. Quality and Bias Assessment

No quality assessment of the studies was performed, as it is not a mandatory procedure in scoping reviews, according to the literature (Arksey & O’malley, 2005; Levac et al., 2010; López-Nieto et al., 2022; Peters et al., 2020).

## 3. Results

The studies included in the present review were conducted in the following countries: the USA (*N* = 9), China (*N* = 1), and Taiwan (*N* = 1). Sample sizes ranged from 4 to 290 participants. The participants ranged in age from 4 to 40 years, encompassing both children and adults, with most studies involving participants aged from approximately 8 to 14 years. One study (Corbett et al., 2025) included participants aged 18–40 years. All selected studies included individuals diagnosed with ASD exclusively. Some studies also included typically developing peers. The characteristics of the included studies are presented in detail in Table A1 in Appendix A.

### 3.1. Design of Included Studies

Among the included studies, seven (7) were identified as randomized controlled trials (Corbett et al., 2014b, 2016, 2017, 2019, 2023, 2025; Ioannou et al., 2020) and four (4) as pilot studies (Corbett et al., 2011; Mehling et al., 2017; So et al., 2019; Wang & Lee, 2020) (Table 2). Furthermore, seven (7) studies applied a control group (Corbett et al., 2016, 2017, 2019, 2023, 2025; Ioannou et al., 2020; So et al., 2019) while others did not (Corbett et al., 2011, 2014b; Mehling et al., 2017; Wang & Lee, 2020). Among those that applied a control group, some used the same intervention after a period (Corbett et al., 2016, 2017, 2019, 2025; Ioannou et al., 2020; So et al., 2019). In contrast, one applied an active control group with a different intervention that had some common elements with the experimental group’s intervention (Corbett et al., 2023) (Table A1).

### 3.2. Diagnostic Tools

The majority of studies that were included in the review used either the 1st edition of the Autism Diagnostic Observation Schedule-Generic (Corbett et al., 2011, 2014b, 2016, 2017; Mehling et al., 2017) or the second edition (Corbett et al., 2019, 2023, 2025; Ioannou et al., 2020; So et al., 2019) to confirm the diagnosis of participants in the autism spectrum (Lord et al., 2000) (ADOS and ADOS-2). To assess the participants’ intelligence quotient (IQ), most of the studies (8 studies) used the Wechsler Abbreviated Scale of Intelligence (WASI & WASI II; Wechsler, 1999, 2011), either the first (Corbett et al., 2014b, 2016, 2017, 2019, 2025) or the second edition (Corbett et al., 2023; Ioannou et al., 2020).

Moreover, two studies (Corbett et al., 2014b, 2025) used the Social Communication Questionnaire (Snow, 2021), a screening tool for ASD, while one study (Corbett et al., 2011) applied the same tool with typically developing children to confirm the absence of autistic traits.

### 3.3. Study Variables and Measurement Tools

Most studies have shown improvements in social and cognitive skills, adaptive functioning, communication abilities, emotional and social perception, interaction and cooperation, as well as reductions in anxiety and stress levels. The variables were assessed through various evaluation tools, questionnaires, and observation protocols, depending on the requirements and goals of each study.

More specifically, social cognition was assessed using the Developmental NEuroPSYchological Assessment (ΝEPSY) and NEPSY II (Korkman et al., 2007). In contrast, one study (So et al., 2019) employed a specially adapted Chinese version of a tool to assess Theory of Mind skills. In one study (Corbett et al., 2025), the Wechsler Memory Scale-Faces (Tulsky et al., 2003) (WMS-F) was used to assess social perception, while another study (Mehling et al., 2017) applied the Penn Facial Emotion Recognition Task (Gur et al., 2002) to measure changes in facial emotion recognition. Another study (So et al., 2019) utilized the Mullen Scale of Early Learning (Lee, 2013) to evaluate motor and cognitive skills. Only one study (Wang & Lee, 2020) did not report the use of a specific diagnostic tool; however, it did conduct a narrative ability screening task before the intervention.

Adaptive social functioning was assessed in three studies using the parent-report measures of the Adaptive Behavior Assessment System (ABAS) (Corbett et al., 2011, 2014b, 2016), while only one study (Corbett et al., 2025) applied the 3rd edition (ABAS-3) (Richardson & Burns, 2005). Additionally, some studies (Corbett et al., 2011, 2014b, 2016) used the Social Responsiveness Scale (SRS) (Constantino, 2002, 2013) to evaluate social functioning, while some others (Corbett et al., 2023, 2025) applied the 2nd edition (SRS-2) of this scale (Constantino, 2012). Two studies (Corbett et al., 2023, 2025) implemented the Contextual Assessment of Social Skills (CASS) (Ratto et al., 2011) as a direct observation tool to record social behavior, while one study (Corbett et al., 2014b) used the Companionship Scale (Bauminger, 2007) to evaluate verbal and non-verbal behavior. In Wang’s (Wang & Lee, 2020) study, the MBV Quantification technique (Albo-Canals et al., 2018) was applied to assess spectrum-related behaviors through video recording. One study (Mehling et al., 2017) reported using the Vineland II Adaptive Behavior Scale (Sparrow, 2011) to track skills through parental reports. Some studies of Corbett et al. (Corbett et al., 2016, 2019, 2023, 2025) also included neurophysiological investigations of the brain’s social functioning using EEG recordings during the Incidental Face Memory task (IFM).

A protocol employed in several studies (Corbett et al., 2014b, 2016, 2017, 2019; Ioannou et al., 2020) is the Peer Interaction Paradigm (PIP), in which researchers analyzed video recordings to assess participants’ interactions with typically developing peers in the playground. The frequency of engaging in Group or Solitary play was also measured.

A central goal in many studies was the evaluation of anxiety and stress levels. To evaluate anxiety and stress levels in individuals with autism, researchers included either questionnaires (e.g., Corbett et al., 2017; Ioannou et al., 2020) or more objective biological indices such as oxytocin and cortisol levels (e.g., Corbett et al., 2011, 2014b, 2017) or both (e.g., Corbett et al., 2014b). Specifically, three (3) studies applied the cortisol sampling collecting protocol (Corbett et al., 2011, 2014b, 2017), often in combination with the Peer Interaction Paradigm. Moreover, two (2) studies (Corbett et al., 2017; Ioannou et al., 2020) utilized the State-Trait Anxiety Inventory for Children (STAI-C) (Spielberger et al., 1973) to assess anxiety as both a state and a trait. In 2011, the study by Corbett et al. (Corbett et al., 2011) employed the Parent Questionnaires Stress Survey Schedule for Persons with Autism and Other Developmental Delays (Groden et al., 2001) to assess stress factors and the Short Sensory Profile (Dunn, 1994) to evaluate sensory sensitivity. This same study also measured oxytocin levels in addition to cortisol sampling. Another study (Corbett et al., 2014b) utilized the Parenting Stress Index (PSI) (Abidin et al., 2006) questionnaire to assess anxiety related to dysfunctions resulting from the dyadic relationship and the parent–child roles.

Lastly, one study (Mehling et al., 2017) evaluated the use of pragmatic language through the Test of Pragmatic Language (Phelps-Terasaki & Phelps-Gunn, 1992, 2007), a tool that measures the ability to use language appropriately in social contexts.

Across the included studies, certain patterns can be observed regarding the domains most responsive to dramatherapy interventions. Measures of social cognition (Theory of Mind tasks, NEPSY) consistently indicated positive changes, suggesting that theater techniques may be particularly effective for fostering these skills. In contrast, tools assessing language abilities (e.g., the Test of Pragmatic Language) demonstrated more modest and variable improvements, suggesting that language-related outcomes may require longer interventions or supplementary approaches. Additionally, anxiety and stress, as measured by both self-reported and parent-reported tools (STAI-C, PSI), revealed mixed findings, while physiological indicators such as cortisol and oxytocin levels remained largely unaffected. Similarly, instruments targeting facial emotion recognition and emotional processing (WMS-F, Penn Facial Recognition Task) showed minimal effects. Taken together, these findings suggest that dramatherapy may exert its most consistent benefits in the domain of social understanding and interaction, whereas outcomes linked to biological stress markers and perceptual processes appear less responsive.

### 3.4. Interventions Using Theater Techniques

Theatre-based interventions in most studies lasted approximately ten weeks (Corbett et al., 2016, 2017, 2019, 2023, 2025; Ioannou et al., 2020; Mehling et al., 2017). One study by Corbett et al. presented an intervention approach lasting three months, while another of her studies, conducted during a summer camp, lasted two weeks. Additionally, intervention in another study (So et al., 2019) lasted twelve weeks, while one study (Wang & Lee, 2020) out of 11 does not report the overall length of the intervention. As for frequency, in most studies (Corbett et al., 2016, 2017, 2019, 2023, 2025; Ioannou et al., 2020; So et al., 2019) theatre-based sessions were conducted once a week. In a 2011 pilot study of Corbett et al. (Corbett et al., 2011), sessions started weekly but gradually increased to three to four times a week, depending on each child’s role demand. In this summer camp study in 2014 (Corbett et al., 2014b), the sessions took place daily, excluding weekends (five times a week for two weeks). In a 2020 study by Wang and Lee (Wang & Lee, 2020), the intervention consisted of five social skills training lessons, though the duration and frequency were not reported.

In many studies (Corbett et al., 2014b, 2016, 2017, 2019; Ioannou et al., 2020), each session lasted four hours. In contrast, in two cases (Corbett et al., 2011, 2025), sessions lasted two hours each. One study (Mehling et al., 2017) implemented one-hour sessions, while another (Corbett et al., 2023) featured three-hour sessions. Finally, in a study (So et al., 2019), the intervention consisted of nine robot-script sessions (three sessions of 45 min each per script), whereas in another study (Wang & Lee, 2020) the session duration is not reported.

Two most recent studies (Corbett et al., 2023, 2025) also involved two days of technical rehearsal and costume fitting. All studies conducted by Corbett et al. (Corbett et al., 2011, 2014b, 2016, 2017, 2019, 2023, 2025), as well as the one by Ioannou et al. (Ioannou et al., 2020), culminated in a public 45 min performance. One study (Corbett et al., 2011) concluded with six public performances, subsequent ones (Corbett et al., 2014b, 2016, 2017, 2019; Ioannou et al., 2020) with two, while the most recent ones (Corbett et al., 2023, 2025) had a single public performance.

For analysis purposes, the studies were categorized into four groups based on the type of program they applied.

#### 3.4.1. Program SENSE Theatre

Most of the studies included in this review were conducted by Blythe A. Corbett and her research team. Corbett is the creator of the innovative theater-based intervention program SENSE Theatre. In total, eight studies implemented the SENSE Theatre approach (Corbett et al., 2011, 2014b, 2016, 2017, 2019, 2023, 2025; Ioannou et al., 2020). Additionally, in 2020, Ioannou et al. conducted a study using the SENSE Theatre program in a group of school-aged children with ASD (Ioannou et al., 2020). In one study (Corbett et al., 2014b), the SENSE Theatre program was implemented in a summer camp, with the duration and frequency of the intervention adjusted while maintaining the program’s core structure and philosophy. Remarkably, in a recent study (Corbett et al., 2025), the SENSE Theatre method was adapted for adults. Clinically trained researchers administered the method. Following the implementation of SENSE Theater, improvements were observed in social cognition, social behavior and functioning, interaction and cooperation, and a reduction in anxiety levels, as well as the maintenance of specific skills, over a two-month period.

#### 3.4.2. Intervention “The Hunter Heartbeat Method”

Τhe Hunter Heartbeat Method was implemented in one study (Mehling et al., 2017) and involved facilitators who were actors selected by a fine arts master’s program. During the intervention, these facilitators modeled the games in the center of a circle, and afterward, they worked one-on-one. The study involved only children with ASD without neurotypical peers as participants. Moreover, it demonstrated improvements in adaptive functioning and pragmatic language, with no significant differences in social cognition (Mehling et al., 2017).

#### 3.4.3. Intervention Using Theater Techniques and Robots

The study of So et al. integrated robots into the intervention through a three-phase approach. Firstly, during the drama performance, two NAO robots performed complete dramas, each playing a character role. Secondly, during the interactive phase, the children assume roles with the robots after watching each drama twice. At this point, it is important to note that the robots provided structured, predictable interactions. Finally, the human generalization phase, in which the children practiced the new skills with a human to demonstrate generalization after the robot’s interaction.

Using this technique with the assistance of robots revealed improvements in narrative ability, with a 40% increase in the use of gestures during storytelling and a 70% success rate in scenario reenactment (So et al., 2019).

#### 3.4.4. Combined Social Play Intervention Using Augmented Reality and Drama-Based Strategies

Wang and Lee (Wang & Lee, 2020) applied a combined social play intervention using augmented reality and drama-based strategies with one child with high-functioning AS and three typically developing children. A total of 5 social training courses were conducted with a special education teacher and a therapist, and the whole process was recorded. Each training involved four children in theater activities, accompanied by the assisted teaching of therapists and observation of social interaction between autistic children and their peers. All children were taught through the theater-based AR social game training system. The teaching content was adapted from the social stories and character situations in the story of “The Wizard of OZ” to provide social training for autistic children and their peers. During the training process, they were combined with theater-based storybooks to guide the game and integrate the theater’s situational props and other characters into the performance. The main aim was to promote autistic children’s pretend play and to guide them in having simple social interactions, and the other three children helped with this. Authors reported improvements in social cognition, reciprocal behaviors, understanding abstract symbols, and metaphorical thinking, as well as increased focus.

To conclude, whereas most people consider drama to be about memorizing texts, the aforementioned techniques and interventions focus on body expression, movement, voice, and interaction. Corbett usually uses her typically developing peers as buddies who also act out the blocking (movement) and lines on video so participants can take that home to review. Most of her studies integrate the participants with typically developing teens and/or adults rehearsing a play that is then performed for a community audience.

### 3.5. Results of the Included Studies

All studies report improvements in most of their dependent variables. No study mentions any adverse outcomes. Three studies (Corbett et al., 2017, 2019, 2023) report that there were no significant differences between the groups at baseline (pre-intervention) and post-intervention on diagnostic and dependent variables.

Specifically, no changes were observed between pre- and post- intervention assessments in facial expression and emotion recognition abilities (Corbett et al., 2011; Mehling et al., 2017), in eye contact (Corbett et al., 2014b) and in non-deictic gestures (So et al., 2019), in social functioning ToM skills in dependent content (Corbett et al., 2019), and behavior variables such as vocal expressiveness, quality of rapport, and social anxiety (Corbett et al., 2023, 2025), as well as in parental anxiety (Corbett et al., 2011) and stress both as measured by oxytocin levels and parental reports (Corbett et al., 2017; Ioannou et al., 2020). Additionally, in three studies, no differences were found between outcomes, such as Play with Equipment (Corbett et al., 2016), Solitary Unsolicited Play (Ioannou et al., 2020), and social cognition assessment (Corbett et al., 2025). In one study (Corbett et al., 2011), oxytocin levels did not differ between the pre- and post-intervention assessments, whereas cortisol levels differed significantly between the beginning of the first and the beginning of the last rehearsal.

In a later study (Corbett et al., 2017), alterations to the play structure did not lead to changes in trait anxiety, and post-intervention cortisol levels during play also showed no changes. In most studies that implemented a two-month follow-up assessment, some results were maintained. However, improvements have been observed in social functioning (Corbett et al., 2016), communication skills (Corbett et al., 2023), social cognition, and behavioral and functional outcomes (Corbett et al., 2025).

### 3.6. Limitations of the Included Studies

A frequently mentioned limitation is the small sample size (Corbett et al., 2011, 2014b, 2017; Mehling et al., 2017; So et al., 2019). In five studies (Corbett et al., 2019, 2023, 2025; Ioannou et al., 2020; So et al., 2019), a lack of diversity and cultural heterogeneity is reported, with the majority of participants being Caucasian. Four studies (Corbett et al., 2011; Mehling et al., 2017; So et al., 2019; Wang & Lee, 2020) did not employ a randomized clinical trial design, while in three studies, the control group was absent (Corbett et al., 2011, 2014b; Mehling et al., 2017). Additionally, a study (Ioannou et al., 2020) notes the absence of an active control group. In two studies (Corbett et al., 2016, 2017), expectancy bias was identified as a potential limitation, as parents or staff aware of the intervention’s goals may have influenced the results. In the most recent studies (Corbett et al., 2023, 2025), the absence of participants with intellectual disabilities is highlighted. Regarding sample composition, one study (Corbett et al., 2011) described a heterogeneous sample, while another (So et al., 2019) noted variability in narrative abilities. In one study (Corbett et al., 2017), the reliability of anxiety measures was questioned due to the use of self-report tools. At the same time, another (Corbett et al., 2016) mentioned the absence of follow-up assessment across all dependent variables and no follow-up beyond the two months. Moreover, two studies were affected by the COVID-19 pandemic—in one case, data were lost (Corbett et al., 2023), and in another, participants were reluctant to attend in-person sessions (Corbett et al., 2025). Additional limitations mentioned individually are: the lack of a requirement for continued attendance in the intervention sessions (Corbett et al., 2011), the absence of external informants, such as teachers, to evaluate the generalization of skills to other contexts (Corbett et al., 2016), the absence of background information on participants or their experience with social skills interventions (Mehling et al., 2017), low engagement in active control group due to the nature of the intervention (Corbett et al., 2023) and the lack of family encouragement to continue therapy (Corbett et al., 2025). Wang’s study (Wang & Lee, 2020) did not report any limitations.

## 4. Discussion

This review aimed to examine and analyze the effects of interventions using theater and dramatherapy techniques exclusively on individuals with ASD. Specifically, the characteristics of the interventions and changes in skills (social, communication, emotional, and cognitive) were documented, and the impact of various intervention forms was highlighted. At the same time, the goal was to enrich the existing literature and support the future design of related studies.

### 4.1. Intervention Characteristics and Implementation

Theater interventions for individuals with ASD are mainly characterized by weekly sessions lasting 1–4 h, with a total duration of approximately 10–12 weeks. They were conducted in specially designed places such as special centers, local schools, universities, and a summer camp, under the guidance of scientific researchers and therapists, as well as specially trained actors and directors. Notably, new technologies were utilized, including video modeling, which research has supported as an effective tool for teaching skills in people with ASD (Delano, 2007; Fragale, 2014), video recording, specially trained robots, and augmented reality (AR). In recent years, studies have highlighted the effectiveness of interventions using AR on children with ASD (Doulah et al., 2023; Koumpouros, 2025; Osadchyi et al., 2020) and emphasized the importance of using robots in therapeutic interventions for this population as a means of sustaining attention (Kumazaki et al., 2020; Ntaountaki et al., 2019).

The studies analyzed in this review were published over the last 14 years, with the majority conducted in the United States (Corbett et al., 2011, 2014b, 2016, 2017, 2019, 2023, 2025; Ioannou et al., 2020; Mehling et al., 2017) with the implementation of SENSE Theatre approach (Corbett et al., 2011, 2014b, 2016, 2017, 2019, 2023, 2025; Ioannou et al., 2020). Of the included studies, only one was conducted in China (So et al., 2019) and one in Taiwan (Wang & Lee, 2020). The dominance of USA-based research and the SENSE Theatre approach is evident and may constitute a publication bias. Moreover, because most studies include Western-centric samples, these findings may not be applicable in Eastern countries. However, the implementation of Theater-based techniques in two Eastern countries reflects a growing interest in this topic. Moreover, the use of innovations in Chinese (e.g., Robot-based play-drama) and Taiwanese studies (e.g., Augmented Reality) reflects a growing international interest in emerging technologies. Such methods highlight the potential for combining traditional dramatherapy with innovative tools to facilitate learning and social interaction in ASD.

### 4.2. Participant Characteristics

Most participants across the included studies were male, which is consistent with the findings from the U.S. Autism and Developmental Disabilities Monitoring Network (ADDM) and DSM-5 (American Psychiatric Association, 2013), which report a 4:1 male-to-female ratio for ASD (Walensky et al., 2023). Additionally, it has been found that Autism Spectrum Disorder manifests differently in girls compared to boys (Duvekot et al., 2017), with many girls adopting “masking” behaviors- actively concealing their symptoms to appear “normal”, which often leads to delayed or no diagnosis at all (Bargiela et al., 2016; Loomes et al., 2017).

Regarding age, most participants were children and adolescents, except for one recent study (Corbett et al., 2025), which was applied only to adults. According to a 2021 systematic review that included 35 studies from 35 countries, the average age of Autism Spectrum Disorder diagnosis is estimated to be around 5 years (Van’t Hof et al., 2021). Although early signs of autism can appear as early as infancy (American Psychiatric Association, 2013), many parents may find it challenging to recognize these signs (Goin-Kochel & Myers, 2005), resulting in an absence or delayed diagnosis. However, early diagnosis and interventions are critical for a child’s future development (Itzchak & Zachor, 2011; Koegel et al., 2014). Theatrical interventions for ages 6–18 primarily aim to enhance social skills, an area where children and adolescents with Autism Spectrum Disorder (Kelly et al., 2018; Knott et al., 2006) struggle, to help them better integrate into society (Mehling et al., 2017).

### 4.3. Social Competence Outcomes

The theater interventions of the present review indicated preliminary positive outcomes in various areas, primarily related to social competence, which is essential for smooth social integration. Specifically, improvements were observed in social cognition, assessed through Theory of Mind evaluations (Martory et al., 2016), as well as changes in social perception, memory ability, and the recognition of emotions and faces (Corbett et al., 2011, 2014b, 2016, 2019; Wang & Lee, 2020). Assessments were primarily conducted using the neuropsychological assessment (Korkman et al., 2007), which provided data on potential changes in social perception. Face memory recognition and proper functioning are fundamental prerequisites, as they activate the recall of socially appropriate information, facilitate the application of Theory of Mind, and contribute to increased participation in group activities (Avery et al., 2016; Corbett et al., 2014a, 2023, 2025). The correlation between acting and the enhancement of social cognition has been demonstrated in both typically developing individuals (Goldstein & Winner, 2012; McDonald et al., 2022) and people with Autism Spectrum Disorder (Guli et al., 2013). Additionally, in Corbett’s studies (Corbett et al., 2019, 2023, 2025), the Incidental Face Memory paradigm was employed to assess face memory, in conjunction with brain activity recordings. Changes in neural signal amplitudes were observed, indicating increased attention to social stimuli. Face memory abilities have been associated with changes in neural responses (Sommer et al., 2023), which are linked to increased attention in social stimuli (Key & Corbett, 2014; Webb et al., 2011).

Improvements were observed in social behavior and interaction, as participants, supported by peers, showed increased engagement in group activities (Corbett et al., 2014b, 2016, 2019; Ioannou et al., 2020; Wang & Lee, 2020). Furthermore, other studies confirm the positive effects of theater interventions, reporting improvements in interaction, cooperation, and social behavior, such as increased eye contact and active participation in group activities (D’Amico et al., 2015; Guli et al., 2013; Reading et al., 2015).

Meanwhile, some studies (Corbett et al., 2014b, 2016, 2025) recorded improvements in social functioning, which include behavioral responses within a social context as an expression of social cognition (Corbett et al., 2016). This domain was extensively assessed using the Social Responsiveness Scale (SRS) (Corbett et al., 2011, 2014b, 2016, 2023, 2025), which focuses on difficulties in social interaction. The findings related to improvements in social functioning are further supported by Guli et al.’s study, which also reported positive outcomes in this area (Guli et al., 2013). Moreover, some studies report improvements in adaptive functioning (Corbett et al., 2014b; Mehling et al., 2017), a broader concept, according to the DSM-5, that encompasses social functioning (American Psychiatric Association, 2013). Both ideas, which often overlap, were also evaluated using the ABAS (Harrison & Oakland, 2015). Moreover, the Hunter heartbeat method, when implemented, showed significant improvements in children’s mood and social engagement at home as parental reports mentioned (Mehling et al., 2017). However, there is a limited amount of literature to compare these findings with other similar studies, as this study was a pilot study. Overall, social functioning improvements were consistently reported across studies and may support that theater is possibly an effective medium to enhance social competence in children and adolescents with Autism Spectrum Disorders.

### 4.4. Anxiety and Stress Outcomes

The included studies reported mixed findings regarding the impact of theatre interventions on anxiety and stress. People with Autism Spectrum Disorder often experience high levels of anxiety. Moreover, as these individuals grow older, their anxiety tends to intensify, as they gradually become more aware of their difficulties and experience the frustration of being unable to socialize (Gotham et al., 2013; MacNeil et al., 2009; van Steensel & Heeman, 2017; White et al., 2009). Some studies included in this review reported a reduction in trait anxiety following the intervention (Corbett et al., 2017; Ioannou et al., 2020). The above may be attributed to the use of self-report measures, which reveal that while children with Autism Spectrum Disorder may struggle to describe how they feel in a particular moment (state anxiety), they are more capable of recognizing overall reductions in anxiety over time (trait anxiety) (Bölte et al., 2008; Simon & Corbett, 2013). These findings are supported by additional studies showing that theater interventions and art therapy result in reduced anxiety levels in typically developing children (Kaplan, 2023; Zhang et al., 2024) and in individuals with Autism Spectrum Disorder (Geiger et al., 2020; Godfrey & Haythorne, 2013; Mpella et al., 2019). Additionally, one study found that reduced anxiety led to increased peer interaction (Corbett et al., 2017). Interventions incorporating peer modeling, structured rehearsal, and performance opportunities may appear effective in promoting self-regulation and coping strategies.

In most interventions, the participation of typically developing peers is highlighted as a means of modeling and support (Corbett et al., 2011, 2014b, 2016, 2017, 2019, 2023, 2025; Ioannou et al., 2020; Wang & Lee, 2020). Corbett’s interventions, utilizing the SENSE Theatre program, were based on the use of trained peers, as this increases opportunities and possibilities for socialization, as well as the generalization of acquired skills (Corbett et al., 2011; Sperry et al., 2010). Many studies reported the benefits of interaction between children with Autism Spectrum Disorder and typically developing peers, while also highlighting the difficulty children with Autism Spectrum Disorder face in observing the behaviors of typically developing peers (Myles et al., 1993; Sperry et al., 2010). For this reason, Corbett’s team incorporated the Peer Interaction Paradigm, in which peers receive specific training aimed at improving Autism Spectrum Disorder-related symptoms (Corbett et al., 2014b), in combination with video modelling, where peers impersonate the role of each participant, to achieve the imitation of gestures and expression, and ultimately, performance of the role (Corbett et al., 2011).

Within the context of SENSE Theatre, the participation of trained peers was combined with the calculation of cortisol levels of people with Autism Spectrum Disorder, aiming to assess the hypothalamic–pituitary–adrenal (HPA) axis, for which cortisol is a primary output and key indicator of stress response (Saxbe, 2008; Young et al., 2021). These interventions employed a saliva sampling protocol to measure cortisol levels as a biological indicator of stress (Hellhammer et al., 2009), which is associated with experiencing stressful situations (Nicolson, 2008). Samples were collected both before and after the intervention, and cortisol levels were compared at the beginning and end of the first, middle, and final sessions. Changes in cortisol levels were found between the start and end of sessions, as well as between the first and middle rehearsal (Corbett et al., 2011, 2017). Additionally, a decrease in cortisol was recorded between the final day and the second playground session with peers, as part of the Peer Interaction Paradigm (Corbett et al., 2014b). In the same study, an increase in cortisol was observed on the first day, which is considered expected, as research suggests that children with Autism Spectrum Disorder exhibit elevated cortisol levels in a playground setting with unfamiliar peers (Corbett et al., 2010; Schupp et al., 2013). In general, cortisol levels vary with age and gender, with younger individuals with Autism Spectrum Disorder often showing lower cortisol levels than older individuals (Corbett et al., 2010; Schupp et al., 2013).

### 4.5. Language and Pragmatic Skills

Improvements were observed in the language domain, with a focus on enhancing pragmatic language (Mehling et al., 2017) and narrative abilities (So et al., 2019). Pragmatic language is associated with the ability to react and respond appropriately in social contexts. These preliminary findings are supported by additional studies, which show that theatrical activities may lead to improvements both in individuals with Autism Spectrum Disorder (Reading et al., 2015; Stratou et al., 2023) and in typically developing individuals (da Cruz et al., 2022), in skills such as taking turns, initiating or participating in conversations, and sharing personal experiences. Although these gains may be preliminary, given the scarcity of studies, they reflect the embedded social-cognitive practice inherent in dramatherapy and may support its use as a context for developing functional communication skills in populations with ASD.

### 4.6. Follow-Up Assessments and Maintenance of Acquired Skills

In some of the interventions presented in this review, follow-up evaluations were conducted after a period (e.g., 2 months or 10 days) to assess the generalization of acquired skills (Corbett et al., 2014b, 2016, 2023, 2025). Specifically, in the study by Corbett, Swain, et al. (Corbett et al., 2014b), a reduction in parental anxiety regarding their child’s condition was observed ten days after the intervention. Parental anxiety, as highlighted in additional research as well, can impact the child’s social functioning (Hall & Graff, 2011). In a follow-up study (Corbett et al., 2016), the positive effects on reciprocal behaviors were maintained, while in another (Corbett et al., 2025), social motivation was sustained. Other studies further confirm the maintenance of these skills following theater interventions (Beadle-Brown et al., 2018; Rahimi Pordanjani, 2021), which report follow-up assessments conducted several months after the intervention, showing either complete retention of improvements or minimal decline in these skills. These exploratory findings indicate that theater-based interventions may have lasting benefits, particularly when they emphasize peer interaction and performance opportunities.

### 4.7. Limitations

This review has several limitations. Methodologically, the inclusion of pilot studies may have introduced potential bias, as the samples were small and not fully representative in some cases, and the grey literature and conference proceedings were omitted. Furthermore, since no formal quality appraisal of included studies was conducted, the reported outcomes should be interpreted with particular caution, especially given the predominance of pilot studies and small samples. Additionally, the search was limited to three databases (Scopus, PubMed, and ERIC) using different search terms, while the references for the included studies were manually searched. However, the databases above are among the most widely used worldwide. Furthermore, only articles with full texts available in English were included, potentially excluding relevant studies published in languages other than English. As for intervention-related limitations, only studies focusing on interventions for people with ASD were selected, following the diagnostic criteria of DSM-5 and its previous version, DSM-IV-TR, which encompasses historic diagnoses of autism, Rett syndrome, Asperger’s syndrome, and pervasive developmental disorders inserted in the search strategy (American Psychiatric Association, 2013). Regarding population characteristics, most participants across the included studies were male. Therefore, this gender bias may have influenced the sample composition of the studies included in this review. Regarding age, most participants were 6–18 years old, except for one recent study that included adults. Two additional limitations, age and gender, could be considered limitations of the studies. Nevertheless, this review addresses a research gap, as there are currently a limited number of studies that specifically focus on the effects of dramatherapy or theater-based interventions on individuals with ASD.

### 4.8. Future Directions

For future research, it is recommended to study theater-based interventions with a larger number of female participants, as the highest reported number of females in a single study was 60 out of 207 participants. Additionally, it is advisable to investigate the effects of theater interventions beyond social competence, such as motor skills, which have not yet been adequately explored. It is noteworthy that all studies excluded individuals with low-functioning Autism Spectrum Disorder, leaving unexamined whether such interventions could also benefit this population. Further research is also recommended on the effects of these techniques in adults. Adults with Autism Spectrum Disorders have higher anxiety levels and face more social challenges than children on the spectrum since social demands are greater at this age. Theatre-based interventions may constitute an important alternative means of increasing social interaction and social cognition in this age group of individuals; however existing findings are highly preliminary since they come from a single study (Corbett et al., 2025). Finally, it is recommended to evaluate the maintenance of acquired skills beyond 2 months to verify their permanence.

## 5. Conclusions

The published studies included in this scoping review are based mainly on the implementation of the SENSE Theatre approach (Corbett et al., 2011, 2014b, 2016, 2017, 2019, 2023, 2025; Ioannou et al., 2020). This fact possibly enables a publication bias. However, the mapping of existing findings suggests that interventions, incorporating theatre-based techniques and dramatherapy within a safe and supportive context, may provoke positive outcomes on social interaction and social cognition in individuals with Autism Spectrum Disorder. These techniques may represent promising practices for clinicians (such as speech therapists, psychologists, and occupational therapists) targeting the amelioration of social interaction and social communication in this population. Moreover, these approaches can be used in education, as theatre-based techniques can be applied in special education contexts, such as role-playing, improvisation, imitation, storytelling, puppets, and masks, to address the difficulties that individuals with Autism Spectrum Disorder often face. The contexts mentioned above, and the application of these methods to them, require interdisciplinary collaboration among various clinicians and therapists. Relevant research often relies on small samples and/or single cases; for this reason, caution is needed when interpreting the results. However, these studies may reveal opportunities to develop a protocol that integrates these interventions into clinical practice for individuals with ASD. Theatre-based and Dramatherapy interventions, therefore, appear to be a promising approach that remains largely unexplored. Nevertheless, because the study was designed to map and summarize the available evidence, these observations should not be interpreted as definitive evidence of intervention efficacy.

## Figures and Tables

**Table 1:** Literature search strategy across databases.

Databases	Search Strategy (23 March 2025)
PUBMED	#1 (ASD OR autism OR autistic OR asperger OR rett OR pervasive OR disintegrative)
	#2 (“dramatherapy” OR “theatre-based techniq*”)
	#3 #1 AND #2
SCOPUS	#1 TITLE-ABS-KEY ((asd OR autism OR autistic OR asperger OR rett OR pervasive OR disintegrative) AND (“dramatherapy” OR “ theatre-based technique”))
ERIC	#1 (autism OR autistic OR ASD OR asperger OR rett OR “pervasive developmental disorder”) AND (dramatherapy OR “drama therapy” OR “theatre-based” OR “theater-based” OR psychodrama OR “role play”)

**Table 2:** Inclusion and exclusion criteria of articles based on the PCC framework.

Domains	Inclusion Criteria	Exclusion Criteria
Participants	This scoping review focuses on articles that examine children, adolescents, and adults with a formal diagnosis of ASD. Studies may or may not include a control group of neurotypical peers.	Studies focus on other neurodevelopmental disorders. Studies that include individuals at risk for ASD without a formal diagnosis.
Concept	The current review tries to identify studies which examine the effects of theatre-based methods in social interactions, social communication, anxiety, and broader psychosocial functioning of individuals with ASD.	Studies not reporting quantitative data.
Context	The study encompasses studies referring to all age ranges and all countries.	Studies no written in English.

## Data Availability

Data are contained within the article.

## Abbreviations

The following abbreviations are used in this manuscript:

SCIT–A	Social Cognition and Interaction Training–Adults
MASSI	Multimodal Anxiety and Social Skills Intervention
PCC	Population, Concept, and Context
PRISMA	Preferred Reporting Items for Systematic reviews and Meta-Analyses
PEERS	Program for the Education and Enrichment of Relational Skills
SENSE	Social Emotional NeuroScience Endocrinology
NEPSY	Neuropsychological Assessment
SRS	Social Responsiveness Scale
SSS	Stress Survey Schedule for Persons with Autism and other Developmental Delays
SSP	Short Sensory Profile
ABAS	Adaptive Behavior Assessment System
PSI	Parent Stress Index
PIP	Peer Interaction Paradigm
STAI-C	State-Trait Anxiety Inventory for Children
CASS	Contextual Assessment of Social Skills
IFM	Incidental Face Memory
ACC	Active Control Condition
TTT	Tackling Teenage Training
WMS-F	Wechsler Memory Scale-Faces
ADOS	Autism Diagnostic Observation Schedule
WASI	Wechsler Abbreviated Scale of Intelligence
SCQ	Social Communication Questionnaire
MSEL	Mullen Scale of Early Learning
ADDM	Autism and Developmental Disabilities Monitoring
DSM	Diagnostic and Statistical Manual of Mental Disorders
HPA	Hypothalamic–Pituitary–Adrenal

## Appendix A

**Table A1:** Characteristics of studies included in the review.

Studies	Design	Sample (n), Country	Participants (Diagnosis/Classification, Age)	Intervention/Control Group	Tools and Methods of Assessment	Study Results
2011 (Corbett et al., 2011)	Pilot	8, USA Unable to complete (*n* = 0)	6 Autism Spectrum Disorder, 2 with pervasive developmental disorder–not otherwise specified Age: 6–17 years old (11 males, 5 females) 8 TD peers	SENSE Theatre, community intervention program/Not applied	Assessment of Theory of Mind via NEPSY Assessment of Social Functioning via SRS Assessment of anxiety via SSS Assessment of Sensory Sensitivity via SSP Assessment of Adaptive Functioning via ABAS Assessment of anxiety via cortisol sampling protocol Collect and assessment of oxytocin levels Pre- and post-intervention assessment	No differences were observed in parent-report evaluations between the groups from pre- to post-intervention. Significant differences were noted in Theory of Mind abilities between pre- and post-intervention assessments. Facial expression recognition did not reach a statistical difference Reduction in cortisol levels from the first to the mid-phase rehearsal. Significant difference in cortisol levels between the beginning of the first and the beginning of the last rehearsal. No differences observed in oxytocin levels between pre- and post-intervention assessments.
2014 (Corbett et al., 2014b)	RCT	16, USA Did not participate (*n* = 4) Unable to complete (*n* = 1)	7 Autism Spectrum Disorder, 4 with pervasive developmental disorder–not otherwise specified, 1 Asperger’s (9 males, 3 females) Age: 8–17 years old	Program SENSE Theatre in summer camp/Not applied	Assessment of changes in Social Perception via NEPSY Assessment of parent–child relationship via PSI Evaluation of Adaptive Functioning in the home environment via ABAS Assessment of Social Functioning through subscales of SRS Observation of Social Behavior (eye contact and active participation) via the Companionship Scale Measurement of anxiety and stress via cortisol sampling protocol Observation of social interaction in the playground via PIP Pre- and post-intervention assessment Evaluation of parental anxiety ten days after the intervention	Increased Social Interaction with peers. No significant difference in eye contact. Significant difference between pre- and post-intervention assessment of face recognition memory and Delayed face memory. Improvement of Social Functioning (social awareness, social cognition). Changes in adaptive skills (Home Living, Self-Care). Significant reduction in parental anxiety in the evaluation ten days after the intervention. Reduction in cortisol levels between the last day of camp and the second playground session.
2016 (Corbett et al., 2016)	RCT	33, USA Unable to complete (*n* = 3)	30 Autism Spectrum Disorder (H.F.) E.G.: 17 (13 boys, 4 girls) C.G: 16 (11 males, 5 females) Age: 8–14 years old 12 trained TD peers	Program SENSE Theatre/Summer Camp	Assessment of Social Functioning via SRS Assessment of Adaptive Functioning via ABAS Observation of Social Interaction via PIP Evaluation of changes in Social Perception (social cognition) via NEPSY Assessment of therapy-related changes in brain social functioning via Incidental Face Memory Task and EEG Pre- and post-intervention assessment Follow-up assessment two months after the intervention	Improvement in Theory of Mind and Social Cognition in the E.G. Following the intervention, the E.G. engaged more frequently in group play compared to the C.G. Significant differences were noted in the amplitude of brain signals and social functioning. No changes between groups in Equipment Play or the amplitude of brain signals in response to non-social stimuli. In the two-month follow-up, the effect on reciprocal communication was maintained, while improvements in social functioning were not sustained.
2017 (Mehling et al., 2017)	Pilot	14, USA Unable to complete (*n* = 0)	14 with Autism Spectrum Disorder Age: 10–13 years old	Intervention “The Hunter Heartbeat Method”/Not applied	Measurement of Adaptive Functioning via parent/caregiver form of Vineland II Assessment of changes in Social Perception via Penn Facial Recognition Assessment of pragmatic language via the Test of Pragmatic Language Evaluation of the intervention and observation of its benefits by parents and participants through a brief social validity questionnaire Pre- and post-intervention assessment	Significant differences in adaptive functioning between pre- and post-intervention assessments Significant differences were reported in pragmatic language between pre- and post-intervention assessments. A substantial majority of participants expressed willingness to participate again in a similar group. Parental reports mentioned improvement in children’s mood and social engagement at home.
2017 (Corbett et al., 2017)	RCT	36, USA Unable to complete (*n* = 3) Did not meet the criteria (*n* = 3)	30 with Autism Spectrum Disorder E.G.: 17 (13 boys, 4 girls) C.G: 13 (11 boys, 2 girls) Age: 8–14 years old 12 trained TD peers	Program SENSE Theatre/Summer Camp	Evaluation of anxiety (as state and trait) and assessment of stress via STAI-C questionnaire and cortisol levels sampling Observation of social interaction and group play in playground sessions via PIP. Pre- and post-intervention assessment	Significant differences in group effect in anxiety as a trait following the intervention. No effects on anxiety as a state Significant differences in cortisol levels between the beginning and end of both the first and mid-phase days of intervention. Increased interaction with peers as a result of reduced anxiety.
2019 (So et al., 2019)	Pilot	26, China Unable to complete (*n* = 0)	26 with Autism Spectrum Disorder E.G.: 13 (11 boys, 2 girls) C.G: 13 (12 males, 1 females) Age: 4–6 years old	Theater-based intervention utilizing robots /Waitlist Control Group	Assessment of narrative ability and theory of mind through specially designed tasks (Chinese version): diverse desire, diverse belief, knowledge assessment, content false belief, explicit false belief, hidden emotion developed by Wellman and Liu (Child Development, March/April 2004, Volume 75, Number 2, Pages 523–541 Scaling of Theory-of-Mind Tasks Henry M. Wellman and David Liu) Pre- and post-intervention assessment Follow-up assessment two weeks after the intervention	The successful enactment of scenarios involving robots and humans occurred over 70% of the total time. Gestures appeared in approximately 40% of interactions with robots and about 44% with humans. The E.G. showed significant improvement compared to the C.G. in the total number of utterances, complex sentences, goal-based stories, stories with cognitive inferences, and the total number of gestures (particularly deictic gestures) per utterance in both immediate and delayed post-treatment assessments. No clear improvements were observed in other types of gestures.
2019 (Corbett et al., 2019)	RCT	102, USA Did not meet criteria (*n* = 15) Unable to complete (*n*= 10)	77 with Autism Spectrum Disorder (H.F) E.G.: 44 (33 boys, 11 girls) C.G: 33 (26 males, 7 females) Age: 8–16 years old	Program SENSE Theatre/Control Group that received the program 6 months later	Measurement of Social Cognition via NEPSY and NEPSY-II Assessment of brain response to external stimuli using the IFM Observation of Social Interaction via PIP Pre- and post-intervention assessment	E.G. showed better performance in the verbal component of the theory of mind. No statistically significant differences in the content-dependent component of the theory of mind After the intervention, E.G. showed a significant increase in brain signals in response to repeated faces compared to single-presentation faces. Following the intervention, E.G. engaged more frequently in cooperative play and verbal interactions during solicited play.
2020 (Wang & Lee, 2020)	Pilot	4 Taiwan Unable to complete (*n* = 0)	1 Autism Spectrum Disorder (H.F.) 3 TD. Age: 6 years old	Combined social play intervention using Augmented Reality (AR) and drama-based strategies/Not applied	Assessment of Social Cognition and Behavior through the MBV Quantification Assessment during the intervention	Improvements in social cognition. Increase in focus and use of interest. Understanding of abstract symbols and symbolic thinking Improvement in reciprocal behaviors.
2020 (Ioannou et al., 2020)	RCT	77 USA	E.G.: 44 C.G: 33 (59 males, 18 females) Age: 8–16 years old	Program SENSE Theatre/Control Group that received the program 6 months later	Observation of Social Interaction in playground sessions through the PIP Assessment of anxiety via the STAI-C questionnaire Pre- and post-intervention assessment	Solicited Play: Following the therapy, E.G. showed increased group play and reduced solitary play during peer interaction, compared to C.G. Unsolicited Play: E.G. demonstrated increased group play. E.G. showed significantly lower levels of anxiety as a trait, compared to C.G. No differences in anxiety as a state.
2023 (Corbett et al., 2023)	RCT	207 USA	207 participants with Autism Spectrum Disorder E.G.: 144 Active Control Group (ACC): 146 (147 males, 60 females) Age: 10–16 years old	Program SENSE Theatre/ACC: TTT	Assessment of brain response to external stimuli using the IFM Observation of Social Behavior via CASS Assessment of Social Functioning via SRS-2 Pre- and post-intervention assessment Assessment ten weeks after the pre-intervention assessment Assessment two months after the intervention	Greater brain response to external stimuli, e.g., compared to C.G. No immediate therapeutic effects or differences between the groups in Social Behavior (Vocal Expressiveness, Quality of Rapport, Social Anxiety) and Social Functioning after treatment. The intervention had an effect in the follow-up assessment two months later on Vocal Expressiveness and Quality of Rapport. In the SRS communication assessment, two months after the intervention, no maintenance of the results was observed. Differences were noted between the pre-intervention SRS communication assessment and the assessment conducted ten weeks later.
2025 (Corbett et al., 2025)	RCT	47 USA	Autism Spectrum Disorder E.G.: 29 C.G: 35 (33 males, 31 females) Age: 18–40 years old	Program SENSE Theatre/Waitlist Control Group, which received the program when all the assessments were complete	Assessment of brain response to external stimuli using the IFM Evaluation of memory and face recognition via WMS-F Observation of Social Functioning via CASS Assessment of Social Functioning via SRS-2 Assessment of adaptive abilities in the home and society environment via ABAS-3 Pre- and post-intervention assessment Follow-up assessment two months later	E.G. showed higher scores in face recognition of repeated faces in the post-intervention assessment compared to C.G. Evaluation of social behavior (Vocal Expressiveness and Quality of Rapport) did not show significant differences between groups after treatment. E.G. showed significant differences in areas of social functioning such as: Communication, Social Communication and Interaction, Social Motivation, and Cognitive Functioning. E.G. showed improvement in adaptive functioning skills compared to C.G. In the follow-up assessment, the skills that were maintained were those related to Social Motivation, while no significant maintenance was observed in social cognition, behavioral, and functional outcomes.

H.F: High Functioning; TD: Typically Developed; SENSE: Social Emotional Neuroscience Endocrinology; NEPSY: Neuropsychological Assessment; SRS: Social Responsiveness Scale; SSS: Stress Survey Schedule for Persons with Autism and Other Developmental Delays; SSP: Short Sensory Profile; ABAS: Adaptive Behavior Assessment System; RCT: Randomized Control Trial; PSI: Parenting Stress Index; PIP: Peer Interaction Paradigm; E.G: Experiment Group; C.G: Control Group; EEG: Electroencephalography; STAI-C: State-Trait Anxiety Inventory for Children; CASS: Contextual Assessment of Social Skills; IFM: Incidental Face Memory; ACC: Active Control Condition; TTT: Tackling Teenage Training; WMS-F: Wechsler Memory Scale-Faces.

**Table A2:** Summary of Assessment instruments and domains assessed across included studies.

Tool	Domain Assessed	Studies (*N*)
ADOS/ADOS-2	Diagnostic confirmation of ASD	9
WASI/WASI-II	Cognitive functioning/IQ	8
SCQ	Autism traits/screening	3
SRS/SRS-2	Social responsiveness/functioning	5
ABAS/ABAS-3	Adaptive behavior (parent-report)	4
NEPSY/NEPSY-II	Social cognition	2
Theory of Mind adapted tool (Chinese version)	Theory of Mind	1
WMS-F	Social perception (faces)	1
Penn Facial Emotion Recognition Task	Emotion recognition	1
Mullen Scales of Early Learning	Cognitive & motor skills	1
Vineland-II	Adaptive behavior	1
CASS	Social skills (direct observation)	2
Companionship Scale	Verbal & non-verbal behavior	1
MBV Quantification (video)	Spectrum-related behaviors	1
PIP (Peer Interaction Paradigm)	Peer interaction/play	5
STAI-C	Anxiety (state & trait)	2
Parenting Stress Index	Parent stress	1
Parent Stress Survey Schedule	Stress	1
Short Sensory Profile	Sensory sensitivity	1
Cortisol/Oxytocin sampling	Stress biology	3
EEG (IFM task)	Neurophysiology of social functioning	3
Test of Pragmatic Language	Pragmatic language	1
